# Supplementary material for: In silico assessment of human Calprotectin subunits (S100A8/A9) in presence of sodium and calcium ions using Molecular Dynamics simulation approach
Source: PLoS One. 2019 Oct 17;14(10):e0224095. doi: 10.1371/journal.pone.0224095 (PMC6797115; doi:10.1371/journal.pone.0224095)
Supplement: S1 Table — (DOCX) [file pone.0224095.s002.docx]

**“S1 Table”.** Summary of the MD runs

| **Duration (ns)** | **Repeat no.** | **Salt concentration** | **The Salt type** | **system** | **No.** |
| --- | --- | --- | --- | --- | --- |
| 100 | 2 | 150 mM | NaCl | A8 | 1 |
| 100 | 2 | 150 mM | CaCl_2_ | A8 | 2 |
| 100 | 2 | 150 mM | NaCl | A9 | 3 |
| 100 | 2 | 150 mM | CaCl_2_ | A9 | 4 |
| 200 | 2 | 150 mM | NaCl | A9/A8 | 5 |
| 200 | 2 | 150 mM | CaCl_2_ | A9/A8 | 6 |
